# Cross-cultural perception of female facial appearance: A multi-ethnic and multi-centre study

**DOI:** 10.1371/journal.pone.0245998

**Published:** 2021-01-22

**Authors:** Rainer Voegeli, Rotraut Schoop, Elodie Prestat-Marquis, Anthony V. Rawlings, Todd K. Shackelford, Bernhard Fink

**Affiliations:** 1 DSM Nutritional Products, Kaiseraugst, Switzerland; 2 Newtone Technologies, Lyon, France; 3 AVR Consulting Ltd, Northwich, Cheshire, United Kingdom; 4 Department of Psychology, Oakland University, Rochester, Michigan, United States of America; 5 Biosocial Science Information, Biedermannsdorf, Austria; 6 Department of Evolutionary Anthropology, University of Vienna, Vienna, Austria; University of Wroclaw, POLAND

## Abstract

Humans extract and use information from the face in assessments of physical appearance. Previous research indicates high agreement about facial attractiveness within and between cultures. However, the use of a narrow age range for facial stimuli, limitations due to unidirectional cross-cultural comparisons, and technical challenges have prevented definitive conclusions about the universality of face perception. In the present study, we imaged the faces of women aged 20 to 69 years in five locations (China, France, India, Japan, and South Africa) and secured age, attractiveness, and health assessments on continuous scales (0–100) from female and male raters (20–66 years) within and across ethnicity. In total, 180 images (36 of each ethnicity) were assessed by 600 raters (120 of each ethnicity), recruited in study centres in the five locations. Linear mixed model analysis revealed main and interaction effects of assessor ethnicity, assessor gender, and photographed participant (“face”) ethnicity on age, attractiveness, and health assessments. Thus, differences in judgments of female facial appearance depend on the ethnicity of the photographed person, the ethnicity of the assessor, and whether the assessor is female or male. Facial age assessments correlated negatively with attractiveness and health assessments. Collectively, these findings provide evidence of cross-cultural variation in assessments of age, and even more of attractiveness, and health, indicating plasticity in perception of female facial appearance across cultures, although the decline in attractiveness and health assessments with age is universally found.

## Introduction

Research documents influences of facial age, health, and attractiveness on impression formation with regard to human female physical appearance [1–7]. Evolutionary scientists suggest that the interest in and sensitivity to female physical appearance is not culturally arbitrary but reflects evolved cognitive mechanisms that motivated successful ancestral human mate selection [6, 8–12]. Because of the links between female fecundity and youth and health [13–19], humans universally ascribe importance to attractiveness, health, and youth in women [3, 20–23].

Apparently variable attractiveness standards across populations have been a topic of systematic research since the observations of Darwin in 1871 [24] and Westermarck in 1891 [25], and social and cultural scientists have advocated against communality in attractiveness assessments across cultures [26]. In this view, population-specific attractiveness standards are a cultural product, acquired by social learning (see Jones and Hill for a discussion [3, 27]. In addition to cultural factors, ecological conditions may influence population differences in attractiveness preferences [28], with the typical finding that male preferences vary less cross-culturally than do female preferences [29].

Research consistently finds that certain characteristics are judged attractive across individuals and cultures [23], suggesting an adaptive function of attractiveness, with external features providing information about biological and social qualities that play a role in sexual selection [30–32]. While most evolutionary-based studies acknowledge cross-cultural consistency in attractiveness assessments (with individual differences larger than differences between cultures) [33, 34], other research suggests that the strategies employed to extract information from faces differ across cultures [35]. One reason for the disagreement may be that environmental settings, and not genes, primarily influence face preferences [36]. In addition, it has been suggested that attractiveness preferences may be population-specific, depending on the ecological conditions and population-specific morphology. In this view, the cross-cultural agreement in face assessments would be higher for the assessment of “unattractive” rather than “attractive” faces [37]. Finally, the majority of reports of cross-cultural attractiveness assessments employed a correlational approach, thus comparing correlations between- and within-cultures based on aggregated rating data. These analyses do not consider random effects of facial stimuli and assessors but rely on *p*-level statistics that may be misleading, especially when sample sizes are low.

The generalizability of conclusions about cross-cultural assessments of facial attractiveness has been a matter of concern, in part because many studies secured facial images and panellists’ ratings from individuals that shared an ethnic background. Another concern is the comparability of findings obtained either from pooling information secured in projects with different foci or from studies that used different equipment and/or protocols. Other studies have investigated cross-cultural assessments of facial appearance by presenting images of individuals of one ethnicity to members of several ethnicities (e.g., [38, 39], or individuals who immigrated to (and live in) a particular country (e.g., [40]). The latter methodology prevents definitive conclusions about the cross-cultural perception of facial appearance, as it is not known whether the immigrants had already adjusted their standards to that of the country to which they immigrated (but see [41] for recent evidence for adaptations to population-specific beauty standards.

Here, we investigate cross-cultural assessments of female facial age, attractiveness, and health in a multi-ethnic and multi-centre study in which female and male individuals identifying with one of five ethnicities (Chinese, French, Indian, Japanese, and South African) judged facial images of women within and across ethnicities. Thus, the current study extends previous research in several ways: i) five ethnicities are considered, concerning both photographed faces and assessors, with stratified random sampling by age (imaged women and assessors) and gender (assessors), ii) imaged women and assessors were selected from an age range of up to ~50 years, iii) the same equipment for imaging and assessment, and the same research protocol, were used in each of five study centres, iv) a mixed-model approach guided analyses of the raw scores (~52,000 judgements per attribute), affording consideration of crossed random effects of facial images and assessors, in addition to fixed effects (face ethnicity, assessor ethnicity, assessor gender). The present study is the first to capitalize on all these aspects together in an effort to advance understanding of cross-cultural perception of female facial appearance.

## Materials and methods

### General methodology

Facial images and rating data were secured in five locations—Guangzhou (China), Lyon (France), New Delhi (India), Tokyo (Japan) and Cape Town (South Africa)—using the same experimental equipment and protocol. Data collection occurred from April 14 to September 6 (image recording), 2019 and from October 22, 2019 to February 1, 2020 (image rating). The study was approved by the Reading Independent Ethics Committee (RIEC), Woodley (U.K.), and the ACEAS Independent Ethics Committee, Ahmedabad (India). All participants provided written informed consent before participating. For images of participants shown in this article, the individuals provided written informed consent for publication.

### Image recording

#### Study sample

Five-hundred-twenty-six women (“participants”) were recruited through local agencies and imaged: Chinese (*n* = 106), French (*n* = 105), Indian (*n* = 100), Japanese (*n* = 100), and South African (*n* = 115). Each sample included participants from five age cohorts (20–29, 30–39, 40–49, 50–59, 60–69 years; *n* = ~ 20 per group, equally distributed around the mean age of the respective group) (Table 1, total sample). Facial skin tone of women varied from darkly pigmented to lightly pigmented, as skin pigmentation correlates with latitude and ultraviolet radiation intensity [42]. However, in some countries the variation in skin tone is greater than in others (e.g., S. Africa) due to genetic variants [43]. According to the Fitzpatrick scale (a widely used photo-type classification tool for UV light sensitivity) [44], with type I = lightest pigmentation, and VI = darkest pigmentation, participants corresponded to the following types: Chinese II-IV, French II-III, Indian IV-V, Japanese II-IV, and S. African V-VI (this assessment was made by skin experts of the study centres).

**Table 1:** Sociodemographic information and skin pigmentation of participants for the total sample and the subsample for the rating study.

			Total sample		Rated sample	
Ethnicity	Fitzpatrick Skin Phototype	Gender	n	Age ± SD [years]	n	Age ± SD [years]
Chinese	II-IV	female	107	45.2 ± 14.1	36	42.6 ± 13.0
French	II-III	female	105	45.0 ± 14.6	36	42.6 ± 13.4
Indian	IV-V	female	100	44.7 ± 14.4	36	42.7 ± 13.2
Japanese	II-IV	female	100	44.9 ± 14.5	36	42.5 ± 13.3
South African	V-VI	female	115	45.7 ± 14.2	36	42.5 ± 13.1

Participants were screened before recruitment and women currently pregnant or lactating, suffering from visible facial pathologies or skin disease, receiving treatment for skin disease, involved in another clinical investigation or having participated in such within the past two months, having facial tattoos or permanent make-up, having topically applied hydroquinone-containing product within the last three months, having a history of facial cosmetic surgery, laser treatment, or application of Botox or hyaluronic acid-based fillers were excluded from participation.

#### Facial imaging

On the day before imaging, no facial cosmetic or dermatological products (including foundation and/or colour products) were allowed. In the evening before the day of imaging, participants could use their regular facial cleanser or soap. On the morning of the day of imaging, participants washed their face with lukewarm water and patted it dry with a soft towel. A technician cleaned the participant’s face by gentle swabbing with a cotton pad soaked with distilled water of ambient temperature and allowed to dry for 20 min. Facial adornment and glasses were removed for imaging. Before taking photographs, participants were acclimatized for 30 minutes at 21±1°C and 45±10% relative humidity.

**Fig 1:**
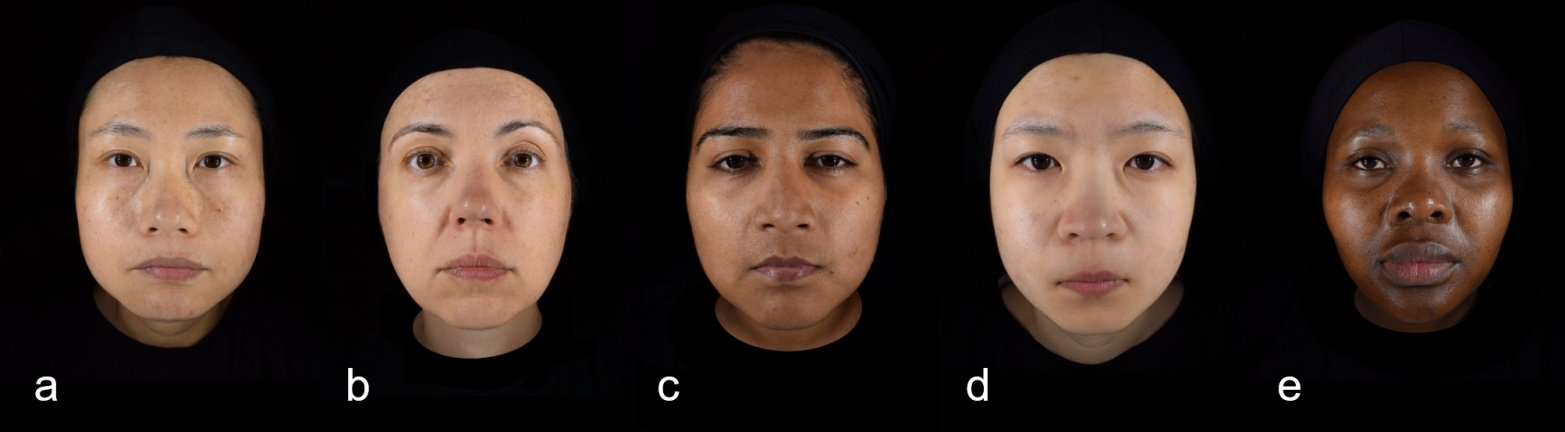
Samples images of female participants for presentation in the rating study. Chinese a), French b), Indian c), Japanese d), and S. African e).

Participants wore identical black hairbands and black capes to cover features that might affect facial assessments (e.g., head hair, chest, or clothes) (Fig 1). Their faces were imaged in frontal view, with eyes open, and with a neutral facial expression using the ColorFace system (Newtone Technologies, Lyon, France). ColorFace captures high-resolution (24 MPs, at a maximum image size of 6000 x 4000 pixels, JPEG file format) full-face images without a chin-rest using an in-built single-lens reflex camera (SLR) camera (Nikon D5300; Nikon Inc., Minato, Japan). Earplugs attached to the stand of the device ensured standardized positioning of participants’ faces, with fixed distance between lens and face. A horizontal reference line connecting the corners of the mouth was displayed on the facial image visualized in real-time on a remote computer, which served as an additional control before image capture. ColorFace uses LED light sources on the left and right sides of the face. System settings were selected to reduce flash intensity and increase light sensitivity of the camera sensor to avoid disturbance of the participant during imaging. For the presentation of the rating study, earplugs were digitally removed from images, eyes were vertically aligned, and visible area of the neck was standardized across images.

### Face ratings

#### Assessors sample

A sample of 600 volunteers (299 females) (“assessors”) participated in the rating study. They were recruited through local agencies in the same locations (and study centres) where the facial images of women were recorded. Participants reported to have lived in the respective location for at least two years. The assessors’ skin photo-types (on the Fitzpatrick scale) matched that of imaged women in each of the five study locations (this assessment was made by skin experts of the study centres). Thus, we had male and female participants of five ethnicities (*n* = 120 per location) (Table 2). Each ethnic group included participants from three age groups (20–34, 35–49, and 50–66 years) with 40 assessors (20 females) per age group. The differences in mean ages between adjacent groups were 15±2 years (all *p*s < 0.001).

**Table 2:** Sociodemographic information and skin pigmentation of assessors in the rating study.

Ethnicity	Fitzpatrick Skin Phototype	Gender	n	Age ± SD [years]
Chinese	II-IV	female	60	42.4 ± 12.6
Chinese	II-IV	male	60	43.2 ± 12.7
French	II-III	female	60	43.7 ± 13.5
French	II-III	male	60	43.7 ± 12.9
Indian	IV-V	female	60	43.1 ± 13.1
Indian	IV-V	male	60	42.4 ± 13.1
Japanese	II-IV	female	60	42.8 ± 12.7
Japanese	II-IV	male	60	43.2 ± 13.2
South African	V-VI	female	59	43.3 ± 13.7
South African	V-VI	male	61	43.3 ± 13.7

#### Procedure

A subset of 180 images (of the initial sample with *n* = 526) was selected for presentation in the rating study (Table 1, rated sample). Before selection of these images, a quality check was performed for suitability of images for inclusion in the rating study. Three raters independently assessed the initial image set on a 4-point scale (1 = *not acceptable*, 4 = *acceptable*) for problems with positioning (e.g., head tilted), visibility of neck, and artefacts due to digital removal of earplugs. Only images considered “acceptable” by all three raters were considered for subset selection (*n* = 382).

**Fig 2:**
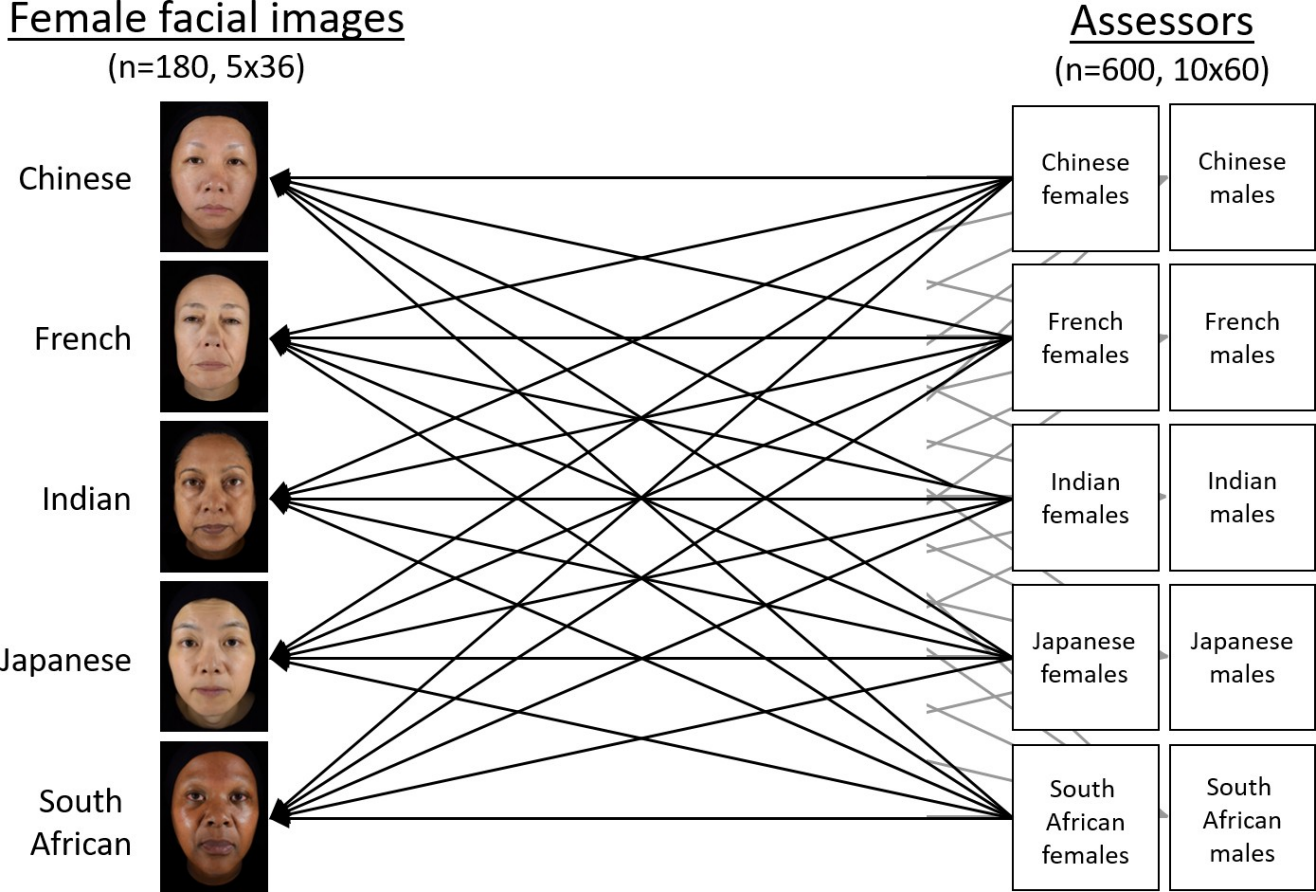
Experimental setup for the face rating. Naïve female and male assessors viewed facial images and provided spontaneous judgments of facial age, attractiveness, and health.

Image selection was randomly stratified for participant/assessor ethnicity, gender, and the three assessor age groups; thus, of the available set of images, 36 images per ethnicity were assigned to female and male assessors of three age groups by considering all possible factor combinations (Fig 2). The images were presented on colour-calibrated, light-shielded, 27-inch LCD monitors (ColorEdge CG277, Eizo, Hakusan, Japan) with faces approximating natural size. Distance of assessor to the monitor during assessment was 50–60 cm. Room conditions during assessment were 21±1°C and 45±10% relative humidity with artificial light only.-

Participants judged the images for age, health, and attractiveness in monadic presentation design (one after the other). Each assessor judged 90 randomly selected facial images per attribute, balanced across age groups (i.e. 270 images, in total). Thus, each image was assessed ~ 300 times and 10 times per subgroup (age group, ethnicity, gender).

Assessments of the three attributes were made in three separate blocks, using web-based software (PhotoScale; Newtone Technologies, Lyon, France). The continuous scales ranged 0–100, with age assessment provided in years, and attractiveness and health assessments ranging “not attractive/not healthy” (0) to “attractive/healthy” (100). Order of blocks was randomized across participants, as was order of images within block. The time for assessment was limited to 3–5 sec. (before the image disappeared) to ensure viewing time was comparable across participants. Breaks of 15 min. were included between blocks to prevent fatigue effects. Statements on the screen and the attributes were created in English and then translated into Mandarin, French, Hindi, Japanese, and Xhosa by native speakers and verified by back-translation.

### Statistical analysis

We performed a series of General Linear Mixed Models (GLMMs), separately for age, attractiveness, and health assessment as dependent variables, and with assessor ethnicity and gender, and participant (“face”) ethnicity as fixed effects (including interactions). Participant and assessor were included as crossed, independent random effects (both *p*s < 0.001). *p*-values of the fixed and interaction effects were corrected for multiplicity using the Benjamini-Hochberg method for control of the false discovery rate [45]. The analysis was performed in *R* [46], using the packages *lme4* [47] and *lmerTest* [48]. We calculated intra-class-coefficients (ICCs) [49] as variance partition coefficients for the mixed effect models. Finally, we aggregated raw scores for age, attractiveness, and health by participant (“face”) and considering assessor ethnicity, and calculated zero-order correlations (Pearson’s *r*) among the attributes for all 25 combinations of assessor ethnicity x face ethnicity (*n* = 36 each).

## Results

Table 3 reports the main effects and interactions of assessor ethnicity, assessor gender and face ethnicity on perception of facial age and Tables 4–6 present descriptive statistics for age, attractiveness, and health assessments, separately for assessor ethnicity and gender, and participant ethnicity.

**Table 3:** Main and interaction effects of assessor ethnicity and gender, and participant (“face”) ethnicity on age, attractiveness, and health assessments.

Factor	Attribute	*F*	DF*	*P*
	Age	10.6	4, 583	<0.001
Assessor Ethnicity (AE)	Attractiveness	1.58	4, 586	0.18
	Health	19.6	4, 587	<0.001
	Age	0.29	1, 583	0.59
Assessor Gender (AG)	Attractiveness	45.2	1, 586	<0.001
	Health	9.87	1, 587	<0.01
	Age	0.94	4, 175	0.44
Face Ethnicity (FE)	Attractiveness	4.17	4, 175	<0.01
	Health	2.41	4, 175	0.051
	Age	2.94	4, 583	<0.05
AE x AG	Attractiveness	4.86	4, 586	<0.001
	Health	0.20	4, 587	0.94
	Age	4.03	4, 51660	<0.01
AG x FE	Attractiveness	6.92	4, 52137	<0.001
	Health	14.7	4, 52295	<0.001
	Age	14.6	16, 51660	<0.001
AE X FE	Attractiveness	181	16, 52132	<0.001
	Health	74.2	16, 52290	<0.001
	Age	1.10	16, 51659	0.35
AE x AG x FE	Attractiveness	4.26	16, 52127	<0.001
	Health	2.93	16, 52286	<0.001

* Calculated with the Satterthwaite method.

**Table 4:** Descriptive statistics of age assessments, separately for assessor ethnicity and gender, and participant (“face”) ethnicity (mean ± SD).

		Face Ethnicity				
		Chinese	French	Indian	Japanese	S. African
Assessor Ethnicity/Gender						
Chinese	F	44.0 ± 13.3	49.6 ± 16.5	47.7 ± 15.9	44.6 ± 14.5	47.0 ± 14.9
Chinese	M	43.4 ±13.0	49.2 ±15.5	47.0 ± 13.9	44.4 ± 13.6	46.0 ± 13.1
French	F	40.3 ± 16.0	44.6 ± 17.0	42.9 ± 16.8	41.7 ± 16.6	43.6 ± 16.4
French	M	42.4 ± 15.3	46.0 +/16.2	45.1 ± 16.3	43.6 ± 16.4	44.9 ± 15.2
Indian	F	42.2 ± 13.3	47.8 ± 15.4	45.3 ± 14.5	43.5 ± 14.3	48.4 ± 14.2
Indian	M	44.3 ± 13.5	49.6 ± 16.0	47.7 ± 14.7	45.4 ± 14.6	49.1 ± 14.9
Japanese	F	42.9 ± 14.8	47.3 ± 17.6	45.1 ± 16.2	43.2 ± 16.5	46.0 ± 15.9
Japanese	M	43.8 ± 14.3	47.7 ± 16.8	45.5 ± 15.4	43.6 ± 15.1	44.9 ± 14.2
S. African	F	45.6 ± 16.1	51.4 ± 17.7	49.8 ± 17.7	46.9 ± 17.1	49.1 ± 16.7
S. African	M	44.3 ± 13.5	49.6 ± 15.2	46.7 ± 15.2	45.1 ± 13.9	46.5 ± 13.7

**Table 5:** Descriptive statistics of attractiveness, separately for assessor ethnicity and gender, and participant (“face”) ethnicity (mean ± SD).

		Face Ethnicity				
		Chinese	French	Indian	Japanese	S. African
Assessor Ethnicity/Gender						
Chinese	F	49.4 ± 18.9	52.8 ± 21.1	48.4 ± 20.3	50.8 ± 19.5	43.5 ± 20.8
Chinese	M	36.3 ± 21.4	38.5 ± 22. 9	34.3 ± 21.5	37.5 ± 21.0	29.3 ± 20.6
French	F	41.5 ± 21.2	45.6 ± 23.0	42.8 ± 21.6	41.6 ± 21.0	43.3 ± 22.6
French	M	31.3 ± 22.0	36.9 ± 25.0	31.7 ± 22.7	31.0 ± 21.5	34.0 ± 23.5
Indian	F	43.6 ± 20.8	46.3 ± 22.6	36.9 ± 18.7	46.3 ± 21.7	25.7 ± 15.9
Indian	M	44.0 ± 19.1	46.2 ± 21.4	37.4 ± 18.1	45.9 ± 19.5	27.2 ± 15.3
Japanese	F	40.4 ± 20.0	51.5 ± 21.2	46.2 ± 21.6	42.8 ± 20.9	44.1 ± 22.0
Japanese	M	31.4 ± 16.3	40.2 ± 20.2	33.6 ± 18.1	33.9 ± 17.6	31.1 ± 18.3
S. African	F	40.0 ± 24.6	37.4 ± 24.7	44.7 ± 26.7	40.4 ± 24.7	39.3 ± 26.0
S. African	M	34.4 ± 22.4	33.9 ± 22.7	37.7 ± 23.7	35.1 ± 23.0	36.3 ± 23.5

**Table 6:** Descriptive statistics of health assessments, separately for assessor ethnicity and gender, and participant (“face”) ethnicity (mean ± SD).

		Face Ethnicity				
		Chinese	French	Indian	Japanese	S. African
Assessor Ethnicity/Gender						
Chinese	F	62.5 ± 17.1	63.4 ± 18.5	60.3 ± 17.9	64.1 ± 17.9	58.0 ± 19.5
Chinese	M	59.3 ± 18.4	59.7 ± 20.0	56.7 ± 19.6	59.9 ± 19.1	56.2 ± 20.4
French	F	67.7 ± 20.2	65.5 ± 22.4	62.3 ± 22.2	67.4 ± 20.6	63.1 ± 21.9
French	M	63.5 ± 18.8	61.4 ± 22.5	58.6 ± 20.9	63.1 ± 20.2	62.8 ± 20.5
Indian	F	55.3 ± 19.4	56.0 ± 20.7	48.4 ± 19.6	58.0 ± 19.2	42.7 ± 19.4
Indian	M	54.2 ± 18.4	53.8 ± 20.4	46.9 ± 17.9	56.3 ± 18.6	42.3 ± 18.0
Japanese	F	61.8 ± 16.9	63.7 ± 18.2	58.1 ± 19.3	65.8 ± 18.1	60.7 ± 18.3
Japanese	M	57.1 ± 17.3	58.8 ± 19.1	55.7 ± 18.3	60.2 ± 17.9	57.2± 18.4
S. African	F	56.4 ± 27.0	51.9 ± 27.3	58.6 ± 27.4	56.0 ± 26.8	53.3 ± 28.3
S. African	M	51.8 ± 23.4	48.4 ± 23.3	52.1 ± 23.9	52.3 ± 23.7	52.1 ± 24.3

### Age

**Fig 3:**
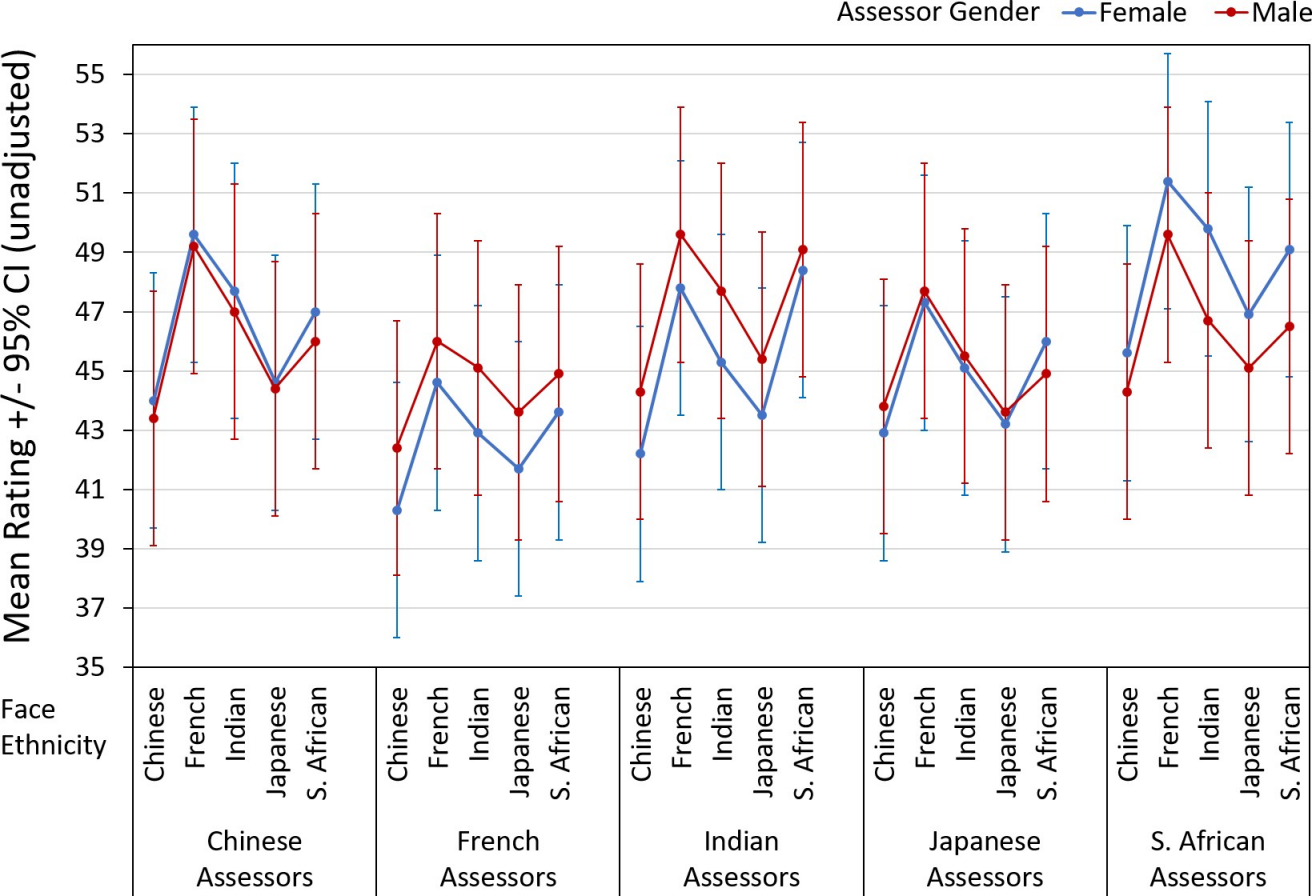
Assessments of facial images, by participant (“face”) ethnicity, assessor ethnicity and gender for age.

Across assessor ethnicities, patterns of facial age perception were similar (Fig 3) across target face ethnicity, with a span of ~5 years (on average) between ethnicities judged youngest (Chinese) and oldest (French). French assessors provided the youngest and S. African assessors provided the oldest age estimations (*p* < 0.001). Neither assessor gender nor face ethnicity showed main effects on age judgements. However, there were interactions of assessor gender with assessor ethnicity and face ethnicity, respectively (both *p*s < 0.05). French women provided younger age estimations than female assessors of other ethnicities, with significant differences between French vs. Chinese and S. African assessors (*p* < 0.01). An interaction was detected for assessor gender with face ethnicity. However, the relevant pairwise comparisons across participants, within females and males, were not significant. The interaction of assessor ethnicity x face ethnicity revealed similar patterns across assessor ethnicities in terms of the rank order of faces. Chinese faces were judged youngest, and French faces oldest, with mean assessments varying across assessor ethnicities (French assessors provided the youngest estimations, and S. African assessors the oldest estimations) (Fig 3). The three-way interaction of assessor ethnicity with assessor gender and face ethnicity on perception of facial age was not significant (Table 3).

### Attractiveness

**Fig 4:**
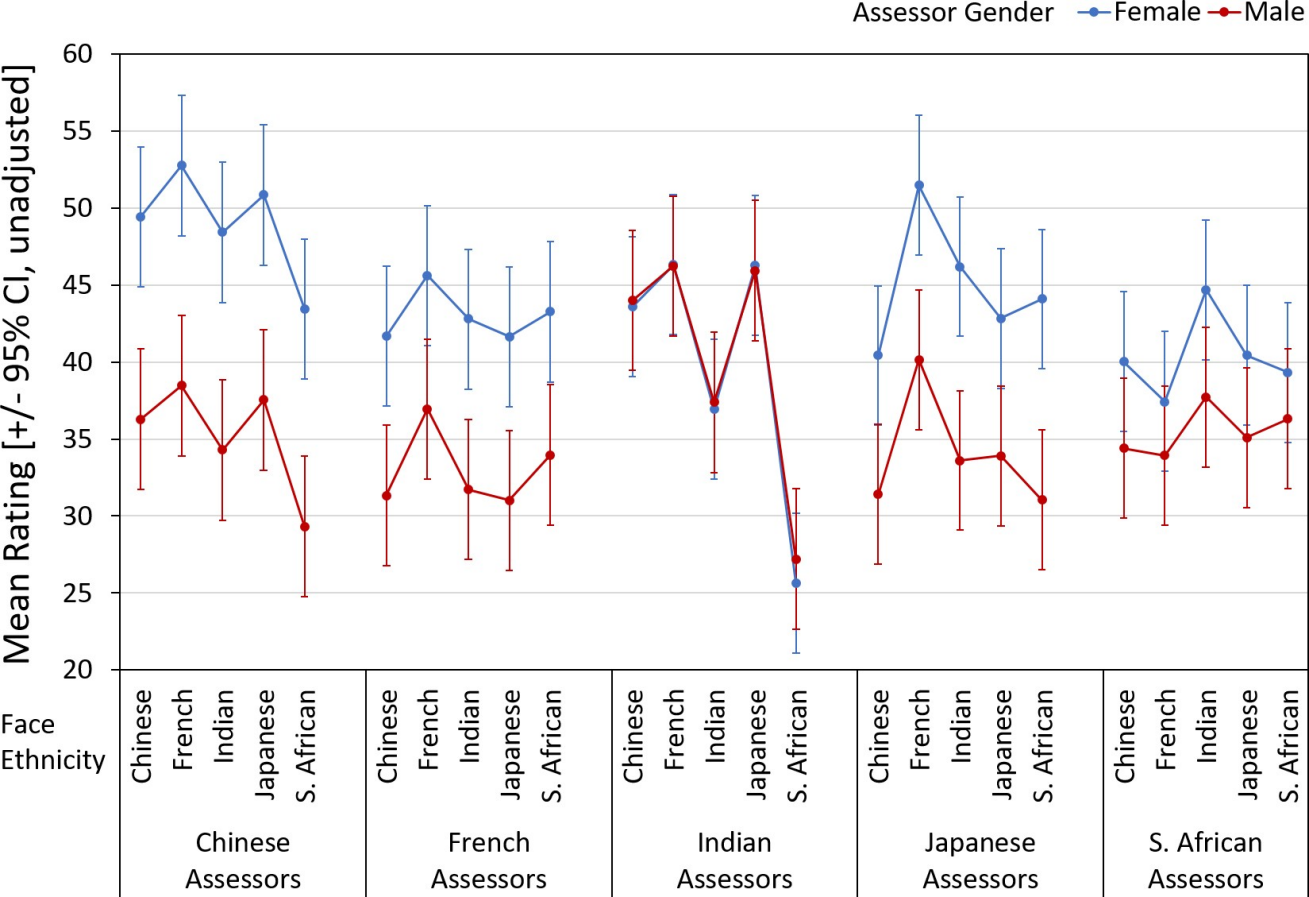
Assessments of facial images, by participant (“face”) ethnicity, assessor ethnicity and gender for attractiveness.

Attractiveness assessments showed considerable variation across assessor ethnicity and face ethnicity (Fig 4), including the interaction of the two effects and a three-way interaction including assessor gender (Table 3). There were main effects of assessor gender (females > males) and face ethnicity (but not assessor ethnicity), with S. African faces judged less attractive than French and Japanese faces (both *p*s < 0.05). Compared with age assessments, the patterns of facial attractiveness judgements were more diverse across assessor ethnicities, with 40 (of 100) significant (*p* < 0.05) pairwise comparisons considering all reasonable comparisons for the interaction of assessor ethnicity x face ethnicity. Chinese, Indian, Japanese, and French assessors judged French women highest in attractiveness (although the pairwise comparisons with those ranked 2^nd^ to 4^th^ were not always significant). S. African assessors, however, provided the highest attractiveness ratings for Indian faces and lowest ratings for French faces, with these two ethnicities showing the only significant difference (Fig 4). Significant gender differences in attractiveness assessments (females > males) were found for all but the Indian and South African assessors. Considering the interaction of assessor ethnicity x assessor gender, eight of 25 pairwise comparisons were significant (all *p*s < 0.05). Chinese women provided the highest attractiveness assessments, followed by Japanese, French, S. African, and Indian assessors (the latter three were significantly different from Chinese assessments at *p* < 0.05). Male attractiveness judgments were highest from Indian assessors, followed by S. African, Chinese, Japanese, and French assessors (with *p* < 0.05 for Indian vs. Japanese and French assessors). The interaction of face ethnicity x assessor gender showed that both female and male assessors judged French faces highest and S. African faces lowest on attractiveness, with females > males (*p* < 0.001) for all pairwise comparisons by face ethnicity. The three-way interaction of assessor ethnicity x face ethnicity x gender indicated that gender differences in mean attractiveness assessments did not generalize across assessor/face ethnicity combinations (with Indian and S. African assessors the exception) (Fig 4).

### Health

**Fig 5:**
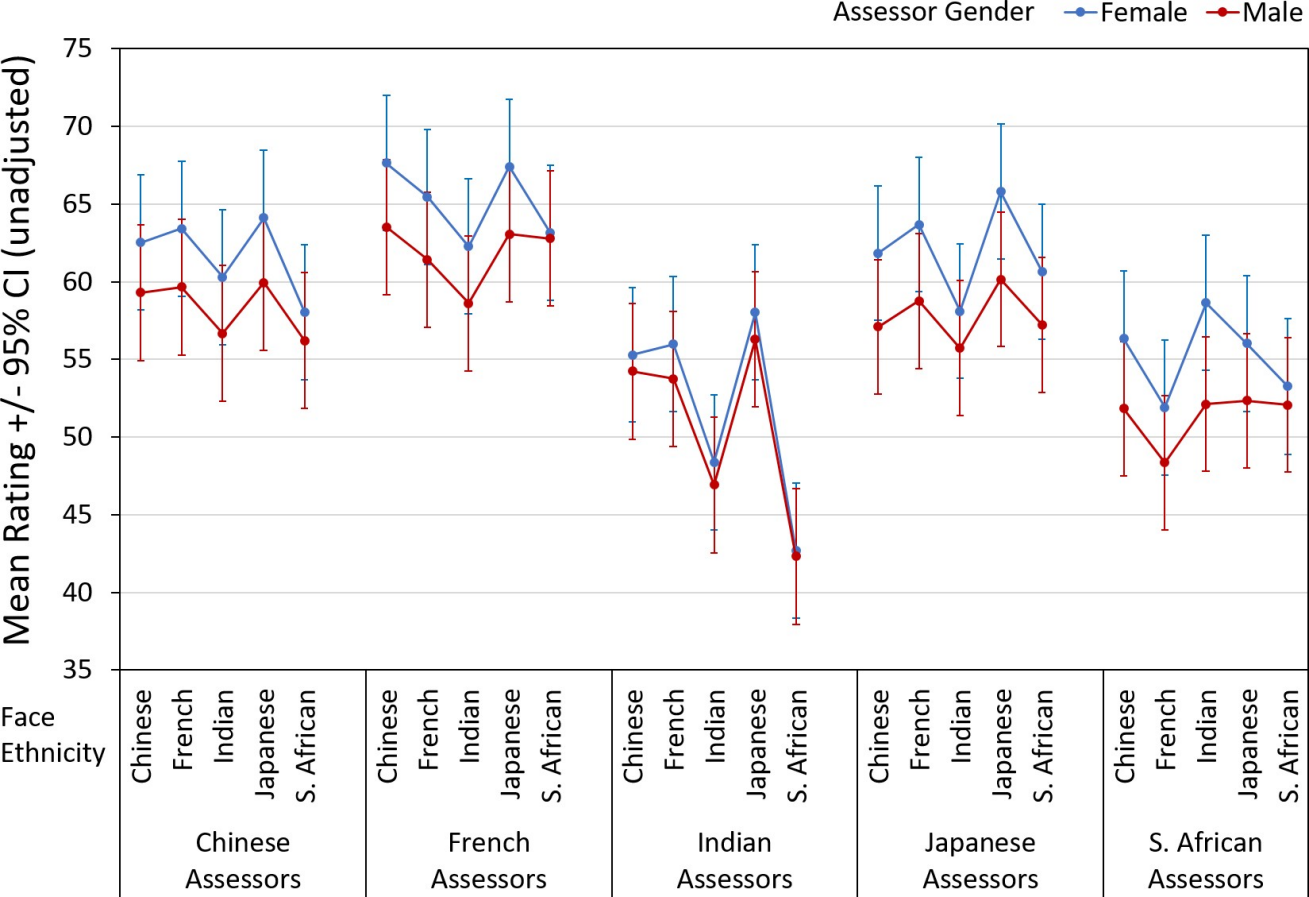
Assessments of facial images, by participant (“face”) ethnicity, assessor ethnicity and gender for health.

Health assessments showed main effects of assessor ethnicity and assessor gender but just failed to reach significance for face ethnicity (*p* = 0.051) (Table 3). Female health assessments were higher than male health assessments. Indian health assessments were lowest, followed by those of S. African, Japanese, Chinese, and French assessors (in that order). Indian and S. African assessments were different from those of Chinese, Japanese and French assessors (*p* < 0.001), but no differences were detected among the latter three (Fig 5). There were interactions of assessor ethnicity x face ethnicity, and face ethnicity x assessor gender (Table 3). The lowest health assessments were of S. African faces by Indian assessors (females and males), these being significantly different from the assessments of other ethnicities’ faces. In contrast, S. African assessors judged Indian faces highest and French faces lowest on health (this being the only significant difference in pairwise tests). Japanese assessors provided higher health ratings of faces of their own ethnicity compared with Indian faces (*p* < 0.05). There were no significant differences (across face ethnicities) in health ratings of Chinese and French assessors. The non-significant finding of assessor ethnicity x assessor gender suggests similar female/male judgements across assessor ethnicities. The three-way interaction of assessor ethnicity x face ethnicity x gender suggests some differences depend on face ethnicity. For example, the rank order of face assessments across ethnicities was the same for female and male Chinese and Japanese assessors, but some differences in comparisons of female vs. male ranking were noted for other assessor ethnicities (albeit n.s. in pairwise comparison across face ethnicities) (Fig 5).

The overall ICCs, i.e. the total variation explained by both participant (“face”) and assessor information, were as follows: age ICC = 0.749, attractiveness ICC = 0.584, health ICC = 0.551. At the participant level (correlations between assessments of the same face), the ICCs were 0.629 (age), 0.130 (attractiveness), and 0.162 (health), and at the assessor level (correlation between judgements by the same assessor) were 0.110 (age), 0.400 (attractiveness), and 0.336 (health).

There were negative correlations of age with attractiveness, ranging -0.680 to -0.908 (all *p*s < 0.001), and health, ranging -0.759 to -0.936 (all *p*s < 0.001), and positive correlations of attractiveness with health, ranging 0.792 to 0.983 (all *p*s < 0.001).

## Discussion

Previous research suggested strong agreement in attractiveness assessments, both within and across ethnicities [23, 40, 50], especially for female attractiveness [29, 51, 52]. The present study used a simultaneous multi-centre, multi-ethnic approach to secure assessments of female facial age, attractiveness, and health and identified both similarities and differences in assessments across ethnicities. Perhaps most importantly, there were (three-way) interaction effects of assessor ethnicity and gender, and participant (“face”) ethnicity for attractiveness and health (but not for age). This suggests that differences in female facial attractiveness and health judgments depend on who judges the face (i.e. assessor ethnicity), which face is assessed (i.e. target ethnicity), and whether the assessor is female or male. There is stronger agreement in facial age assessments than in attractiveness and health assessments.

Intra-class correlations (ICCs) corroborate the findings of diversity in cross-cultural face assessments; the ICC for age assessments was higher than for attractiveness and health assessments, suggesting greater agreement for the former than the latter assessments. Inter-correlations of female facial age, attractiveness, and health assessments were large and in the direction predicted by evolutionary approaches to female appearance (see for review Grammer et *al*. [9], Rhodes [4], and Thornhill and Gangestad [12]), suggesting a strong relationship of attractiveness with health, and a decline in these qualities with age [3, 17, 53]. Collectively, the findings of the present study suggest greater cross-cultural variation in assessments of female facial appearance than indicated in previous research, especially in attractiveness and health assessments.

Recent research reported disagreement among individual facial attractiveness judgements, highlighting the importance of determining how these preferences vary among individuals [54, 55]. Perhaps most relevant for cross-cultural comparisons is the assumed importance of certain facial characteristics in a given society as derived from the study of another society. Facial characteristics investigated in previous studies (e.g., symmetry, averageness, sex-typical features) may not contribute substantially to judgements of facial attractiveness [56–58] or health [59], but even if they do, the contribution of these features may vary across societies depending on environmental conditions [60, 61] or sociocultural settings [62, 63]. Zhang et *al*. [57] in a data-driven (as opposed to theory-driven) approach detected cross-cultural differences in face preferences not apparent in studies using theory-driven approaches, leading to the conclusion that Chinese and British “White” participants used face information in different ways (i.e. they focused on different features) (see also Kleisner et *al*. [64]). Similar conclusions were derived from the findings of eye-movement patterns of Western and East Asian participants, suggesting that cultural background shapes visual environment affordance [35]. Coetzee et *al*. [65] investigated attractiveness assessments of White Scottish and Black S. African students for own- and other ethnicity faces. Black S. African raters relied more heavily on colour cues in their assessments of Black African female attractiveness, whereas White Scottish judges relied more heavily on shape cues in their assessments. The researchers concluded that although there was evidence for the universality of facial attractiveness assessments, the ethnicity of the target face moderated this agreement, i.e. agreement on European faces was higher than on African faces (possibly due to a difference in familiarity with other-ethnicity faces).

In the present study, the female participants (imaged women) were recruited in major cities. We might assume that contact with other ethnicities is considerable. Coetzee et *al*. [65] stated for S. Africans, for example, there is variation across samples in terms of familiarity with other ethnicities’ facial appearance. However, this alone cannot explain the variation in the facial assessments across ethnicities in our findings. The patterns of age assessments are similar across ethnicities, for both face ethnicity and assessor ethnicity. If assessors of one ethnicity were unable to accurately assess facial appearance of other ethnicities because of unfamiliarity with the variation in morphology, the patterns of age assessments across ethnicities should be more diverse than was the case (although there were differences in mean age assessments). Age-related changes in facial morphology (in terms of shape) and visible skin condition both play a role in age assessments. Yet the relative contribution of these features to age perception may be different across ethnicities depending, for example, on the visibility of skin colouration cues. In lightly pigmented skin, unevenness may be more detectable than in darkly pigmented skin. In the present study, our focus was on the investigation of cross-cultural differences (or similarities) in perceptions of female facial appearance. Thus, we did not quantify facial morphology and/or skin condition. As such, the possibility of cross-cultural variation in the relative importance of these components for age assessments remains to be investigated.

Attractiveness and health assessments showed greater variation across ethnicities, with some large differences associated with face and assessor ethnicity, in addition to gender differences. Perhaps most conspicuous in the pattern of cross-cultural variation in facial attractiveness and health is the low assessments of S. African (and Indian) women (and the absence of a gender difference) made by Indian assessors. This may reflect the influence of socio-cultural factors, namely “colourism” (i.e. a preference for lighter skin colour, possibly dating to colonialism) [66] (but see Wagatsuma, 1967 [67]), on face perception, as darkly pigmented skin in India is perceived negatively, partially due to the hierarchical caste system [68, 69]. Similar “colourism” has been reported for S. Africa where lighter-skinned migrants have been treated more positively than darker-skinned migrants [70]. In the present study, S. African assessors judged French faces lowest and Indian faces highest on attractiveness.

Many additional factors might contribute to cross-cultural differences in attractiveness assessments, including environmental settings [29, 71–73] and measures of national health [28, 39], along with variation within- and between assessors (e.g., hormonal fluctuations), which have been reviewed elsewhere [4, 12, 30, 74, 75] (but see Jones et *al*. and Marcinkowska et *al*. [76, 77]). There is consensus that certain facial cues relate to female age and health, both of which correlate with female fecundity and reproductive potential [9, 13, 15, 78]. From an evolutionary perspective, one might assume that these relationships are found universally, and the evidence from industrialized and pre-industrialized societies suggests that this is the case. However, this universality does not preclude variation in the strength of associations across ethnicities. Our findings of cross-cultural variation in perceptions of female facial appearance do not challenge the evidence that certain facial cues provide information about an individual’s mating-related quality. We document negative correlations between age and attractiveness/health, and a positive correlation between attractiveness and health for every combination of face ethnicity and assessor ethnicity. The relative size of effects and the mean assessments may differ across cultures because of differences in environmental conditions, socio-cultural factors, and other variables that contribute to individual differences (see for a review, Pisanski and Feinberg [79]). Nevertheless, the biological blueprint nature uses to convey certain information about an individual’s quality may be the same for all humans [9].

Many studies investigating human physical attractiveness include a statement on the stability of attractiveness ratings across ethnicities (“strong cross-cultural agreement”). However, there is concern about the validity of this statement [54, 55, 80, 81]. The findings of the present study corroborate the presence of differences in the assessment of female facial appearance, depending on the ethnicity of the face and the ethnicity and gender of the assessor. These cross-cultural differences in face assessments are evident especially in attractiveness and health ratings, at least in samples of industrialized and industrializing countries. Previous research reporting differences in face preferences of industrialized vs. pre-industrialized societies [82, 83] suggested that visual experience with facial cues may account for the effect (but see Danel et *al*. [80]). We suggest that visual experience with faces of other ethnicities alone cannot explain our findings. Rather, our findings may be explained through a combination of ethnocentrism [84, 85] and other effects that emerge from different socio-cultural settings. However, the variation in patterns of assessments of female facial appearance may also reflect evolved preferences expressed in response to environmental settings that contributed to the development of plasticity in the perception of female facial appearance across cultures. Future studies should i) quantify cross-cultural variation in facial morphology and visual skin condition, and disentangle the relative impact of these components on face ratings, and ii) consider the influence of ethnocentrism and stereotyping in cross-cultural (facial) assessment, in addition to effects motivated by human sexual psychology. For example, face research has successfully applied geometric morphometrics in the assessment of facial shape variation in samples of industrialized and non-industrialized societies in relation to physical capacity and/or perception (e.g., Butovskaya et *al*., Fink et *al*., Schaefer et *al*., and Kleisner et *al*. [86–89]). Similarly, objective measures of skin color and the evenness of skin tone correlate with assessments of facial age, attractiveness, and health [90, 91]. The application of these technologies in the current multi-ethnic and multi-centre study would take the study findings to the next level by investigating features that predict cross-cultural variation in face assessments.

Although the high level of standardization of facial imaging and assessment protocols is a strength of the current study, we contend that it could be realized only in cooperation with local study centres in major cities. The collection of similar stimuli and information from members of small-scale societies in anthropological fieldwork remains challenging. Therefore, evidence from studies that have investigated face assessments cross-culturally should be considered with caution regarding the comparability of study findings. This includes questions about influences from (Western) media shaped face perception, which can be assumed to be present in all population samples of the current study. France, for example, is a global leader in the cosmetics business, and French cosmetic products are highly regarded especially in China and Japan, possibly leading to stereotypic and higher assessments of French women compared to women of other ethnicities. We suggest that studies investigating cross-cultural agreement in face perception and the reasons for geographical variation need to quantify socio-cultural stereotypes (e.g., Choi et *al*. [92] in inter-population perception in addition to securing objective measures of biological variation in facial appearance.
